# Neuronal ER-Signalosome Proteins as Early Biomarkers in Prodromal Alzheimer's Disease Independent of Amyloid-β Production and Tau Phosphorylation

**DOI:** 10.3389/fnmol.2022.879146

**Published:** 2022-05-05

**Authors:** Fátima Mesa-Herrera, Raquel Marín, Eduardo Torrealba, Guido Santos, Mario Díaz

**Affiliations:** ^1^Laboratory of Membrane Physiology and Biophysics, Department of Animal Biology, Edaphology and Geology, Biology Section, Science School, Universidad de La Laguna, San Cristóbal de La Laguna, Spain; ^2^Laboratory of Cellular Neurobiology, Department of Basic Medical Sciences, Medicine Section, Health Sciences School, Universidad de La Laguna, San Cristóbal de La Laguna, Spain; ^3^Associate Research Unit ULL-CSIC “Membrane Physiology and Biophysics in Neurodegenerative and Cancer Diseases”, University of La Laguna, San Cristóbal de La Laguna, Spain; ^4^Instituto Universitario de Neurociencias (IUNE), Universidad de La Laguna, San Cristóbal de La Laguna, Spain; ^5^Department of Neurology, Hospital Universitario de Gran Canaria Dr. Negrín, Las Palmas de Gran Canaria, Spain; ^6^Systems Biology and Mathematical Modelling Group, Department of Department of Biochemistry, Microbiology, Cell Biology and Genetics Biology Section, Science School, Universidad de La Laguna, San Cristóbal de La Laguna, Spain; ^7^Department of Physics, Faculty of Sciences, Universidad de La Laguna, San Cristóbal de La Laguna, Spain

**Keywords:** Alzheimer's disease, mild cognitive impairment, subjective memory complaints, cerebrospinal fluid, estrogen receptors (ER), ER-associated signalosome, lipid rafts, multivariate biomarkers

## Abstract

There exists considerable interest to unveil preclinical period and prodromal stages of Alzheimer's disease (AD). The mild cognitive impairment (MCI) is characterized by significant memory and/or other cognitive domains impairments, and is often considered the prodromal phase of AD. The cerebrospinal fluid (CSF) levels of β-amyloid (βA), total tau (t-tau), and phosphorylated tau (*p*-tau) have been used as biomarkers of AD albeit their significance as indicators during early stages of AD remains far from accurate. The new biomarkers are being intensively sought as to allow identification of pathological processes underlying early stages of AD. Fifty-three participants (75.4 ± 8.3 years) were classified in three groups as cognitively normal healthy controls (HC), MCI, and subjective memory complaints (SMC). The subjects were subjected to a battery of neurocognitive tests and underwent lumbar puncture for CSF extraction. The CSF levels of estrogen-receptor (ER)-signalosome proteins, βA, t-tau and p-tau, were submitted to univariate, bivariate, and multivariate statistical analyses. We have found that the components of the ER-signalosome, namely, caveolin-1, flotilin-1, and estrogen receptor alpha (ERα), insulin growth factor-1 receptor β (IGF1Rβ), prion protein (PrP), and plasmalemmal voltage dependent anion channel 1 (VDAC) could be detected in the CSF from all subjects of the HC, MCI, and SMC groups. The six proteins appeared elevated in MCI and slightly increased in SMC subjects compared to HC, suggesting that signalosome proteins undergo very early modifications in nerve cells. Using a multivariate approach, we have found that the combination of ERα, IGF-1Rβ, and VDAC are the main determinants of group segregation with resolution enough to predict the MCI stage. The analyses of bivariate relationships indicated that collinearity of ER-signalosome proteins vary depending on the stage, with some pairs displaying opposed relationships between HC and MCI groups, and the SMC stage showing either no relationships or behaviors similar to either HC or MCI stages. The multinomial logistic regression models of changes in ER-signalosome proteins provide reliable predictive criteria, particularly for the MCI. Notably, most of the statistical analyses revealed no significant relationships or interactions with classical AD biomarkers at either disease stage. Finally, the multivariate functions were highly correlated with outcomes from neurocognitive tests for episodic memory. These results demonstrate that alterations in ER-signalosome might provide useful diagnostic information on preclinical stages of AD, independently from classical biomarkers.

## Introduction

There is considerable epidemiological evidence supporting the notion that Alzheimer's disease (AD) has a long preclinical period (Dubois et al., 2016; Haaksma et al., 2017; Jack et al., 2018). Mild cognitive impairment (MCI) is often considered a precursor stage of AD-type dementia (DeCarli, 2003; Haaksma et al., 2017; Jack et al., 2018). This syndrome is characterized by the memory impairments and its evolution to AD occurs at a rate of 10–15% per year, with 80% conversion by the sixth year of follow-up (DeCarli, 2003; Dubois et al., 2016). Since AD is the most prevalent neurodegenerative disorder in the elderly, and is preceded by a long phase of neuropathological changes and cognitive decline before it is diagnosed, it is paramount in the development of novel biomarkers which allow monitoring disease progression from MCI to AD. In fact, the current strategies for biomarkers searching in AD include neuroimaging techniques and biochemical analysis of different fluids, mainly cerebrospinal fluid (CSF) and blood (Bloudek et al., 2011; Zhang et al., 2014; Blennow, 2017; Khoury and Ghossoub, 2019; Del Prete et al., 2020). The CSF levels of β amyloid (βA) peptides, total tau (t-tau), and phosphorylated tau (p-tau) have been accepted as molecular biomarkers in the diagnosis of AD (Anoop et al., 2010; Blennow et al., 2015; Blennow, 2017; Khoury and Ghossoub, 2019). However, these classical biomarkers do not allow monitoring the evolution of the disease from prodromal stages (Forlenza et al., 2010; Dubois et al., 2016; Del Prete et al., 2020) and the recent reports have demonstrated their lack of specificity (Riemenschneider et al., 2002; Bibl et al., 2006; Hyeon et al., 2015; Abu Rumeileh et al., 2017). Thus, great efforts are currently being endeavored in the pursuit for novel biomarkers different from those related to βA generation and tau phosphorylation, for the identification of prodromal AD stages. Such novel strategies focus on the different pathological processes underlying or related to AD, including neuroinflammation (Kinney et al., 2018), synaptic dysfunction (Marsh and Alifragis, 2018), connectomics (Yu et al., 2021), abnormal neuronal signaling and altered neurotransmission (Reddy, 2017), neurolipid alterations (García-Viñuales et al., 2021), metabolic impairment (Ferrer, 2009), oxidative stress (García-Blanco et al., 2017), and stress resistance (Lu et al., 2014), protein aggregation and/or degradation (Tsuji and Shimohama, 2002), nerve cell injury and death mechanisms such ferroptosis (Zhang et al., 2021). In recent years, several non-amyloid and non-tau -related candidates have been proposed, these including the synaptic protein neurogranin that seems specific for AD and to predict future rate of cognitive deterioration (Thorsell et al., 2010; Blennow, 2017), TDP-43, a marker of protein aggregates which is correlated with clinical and neuropathology features indexes of MCI and AD patients (Tremblay et al., 2011) and neurofilament light chain, a marker of neurodegeneration (Mattsson et al., 2017; Gaetani et al., 2019; Zetterberg and Blennow, 2021), amongst others. However, although these potential biomarkers have been associated with AD and even to MCI (Thorsell et al., 2010; Tremblay et al., 2011; Gaetani et al., 2019), they do not exhibit specificity for AD. For instance, TDP43 has been reported as a biomarker for the frontotemporal lobar degeneration (FTLD), amyotrophic lateral sclerosis (ALS), and limbic-predominant age-related TDP-43 encephalopathy (LATE) (Steinacker et al., 2008; Majumder et al., 2018; Nelson et al., 2019).

The signalosomes are multimolecular complexes formed by specific subsets of proteins which interact physically and participate in cellular responses often involving signaling events present in a number of cell types, including neurons (Marin, 2011; Hicks et al., 2012; Meininger and Krappmann, 2016; DeBruine et al., 2017; Dubiel et al., 2020; Colozza and Koo, 2021; Xu and Lei, 2021). The signalosomes are organized as multimolecular clusters whose components interact dynamically following spatiotemporal patterns (Hundsrucker and Klussmann, 2008; Wu and Fuxreiter, 2016; Kandy et al., 2021; Zaccolo et al., 2021). Our recent research in neuronal cells demonstrate that neuronal lipid rafts are the locus of a particular signalosome, the estrogen-receptor (ER)-signalosome, formed by a complex set of factors involved in cellular signaling and neuronal survival (Marin et al., 2009, 2012; Marin, 2011; Marin and Diaz, 2018). The main components of ER-signalosome are pro-survival receptors ERα (estrogen receptor α) and IGF-1Rβ (insulin-like growth factor 1 receptor β), scaffold proteins caveolin-1 and flotillin, prion protein (PrPc), pl-voltage dependent anion channel 1 (VDAC) (a plasmalemmal form of VDAC1) and ionotropic NMDAR and metabotropic mGluR5 glutamate receptors (Marin et al., 2008, 2009; Ramírez et al., 2009; Alonso and Gonzalez, 2012; Díaz and Marin, 2021). Further, the current evidence indicates that the neuronal ER-signalosome likely includes signal transducers such as monomeric G-protein, Ras, and tyrosine kinases such as Raf-1 involved in MEK/ERK signaling for ERα-mediated neuroprotection (Marin et al., 2003, 2005; Guerra et al., 2004). An emerging concept is that the potential disruption of lipid rafts–resident signalosomes, as a consequence of factors affecting the homeostasis of lipid rafts, contributes to the etiology of AD (Marin et al., 2013; Canerina-Amaro et al., 2017). A striking feature of neuronal ER-signalosome is that proapoptotic protein pl-VDAC shares a common cluster with survival factors ERα and IGF-1Rβ within lipid rafts (Marin et al., 2009; Ramírez et al., 2009; Alonso and Gonzalez, 2012). Our initial observations revealed that under resting conditions pl-VDAC remains inactive through the modulation of its phosphorylation state, a process that appears to be ERα-mediated (Herrera et al., 2011a,b; Canerina-Amaro et al., 2017; Marin and Diaz, 2018). However, at least in cultured neurons, induction of amyloid toxicity triggers the dephosphorylation of the channels and eventually cell death (Marin et al., 2007; Herrera et al., 2011a,b; Fernandez-Echevarria et al., 2014). The mechanistic relevance of these observations is that VDAC might switch the normal functionality of the ER-signalosome as cell survival mechanism into cell death upon changes in its phosphorylation/dephosphorylation status (Marin et al., 2007; Herrera et al., 2011a; George and Wu, 2012). In agreement, the previous studies carried out in our laboratory have revealed the disruption of the molecular complex formed by ERα, pl-VDAC, and caveolin-1 in brain cortex lipid rafts at late stages of AD (Marin et al., 2007; Ramírez et al., 2009). In cortical raft fractions isolated from AD brains, pl-VDAC appears mostly dephosphorylated as compared to age-matched control brains (Canerina-Amaro et al., 2017). Our preliminary results demonstrate the presence of ERα, IGF-1Rβ, caveolin-1, flotillin, PrPc, pl-VDAC, and NMDAR in the CSF of patients with advanced AD. The question then arises on whether changes in ER-signalosome proteins occurs at early stages of AD-type neurodegeneration and that whether these changes might be followed in the CSF.

In this study, we have aimed to determine the potential presence of signalosome proteins in the CSF of healthy subjects and patients with prodromal stages of AD. We have undertaken not only the identification of ER-signalosome proteins but also their quantitative relationships as well as their correlation with classical AD biomarkers. The results indicate that ER-signalosome might well be potential biomarkers of AD progression at preclinical stages. The dynamic changes of these potential biomarkers in the CSF could help to understand the biochemical processes underlying the early pathology associated with AD.

## Materials and Methods

### Participants

The participants were consecutively recruited at Hospital Universitario de Gran Canaria Dr. Negrin in accordance with ethical principles stated in the Declaration of Helsinki, as well as with approved national and international guidelines for human research. The study was reviewed and approved by Ethical committee of Hospital Universitario de Gran Canaria Dr. Negrin. All participants provided their written informed consent to participate in this study. The inclusion criteria were consecutive outpatients at the Neurology service, older than 59 years, able to read and write, consulting for memory complaints.

### Classification of the Subjects

The healthy controls (HC) were recruited from the community and the orthopedic surgery scheduled for a CSF study. The control group was composed of patients with either clinically or pathologically defined alternative diagnosis and not included cases other neurological diseases. The control cases did not present biomarker profiles indicating the presence of a neurodegenerative disease.

Subjective memory complaints (SMC) group includes the patients who refer for the cognitive complaints but had a normal performance in a full neuropsychological evaluation without impact in daily living.

The MCI group was formed by patients who satisfied the criteria from the report of the MCI working group of the European Alzheimer's disease Consortium (EADC) (Portet et al., 2006). The global deterioration scale (GDS) was used to classify the global deterioration (Reisberg et al., 1988) into HC participants (neither cognitive complaints nor dementia, GDS1); SMC, (normal performance in the neuropsychological battery, GDS2); MCI (GDS3) with low performance in at least one cognitive function (patients more than 1.5 standard deviations below the mean, according to the Spanish data from NORMACODEM and NEURONORMA Studies (Peña-Casanova et al., 2005, 2009a,b,c,d; Casals-Coll et al., 2014) in not demented patients.

The exclusion criteria were the suspected focal or diffuse brain damage due to clinical conditions different to AD; uncontrolled systemic diseases or delirium in the last 30 days; history of drug addiction or alcoholism, being under treatment for AD; history of major depression or being under treatment with two or more antidepressants; more than one dose per day of benzodiazepines; severe perceptive or motor disorders. The subjects with dementia were excluded based on the assessment by Blessed Dementia Rating Scale (BDRS) and Instrumental Activities for Daily Living (IADL) (BDRS part A was more than 1.5 and IADL was <6 for women and <5 for men) (Blessed et al., 1968; Peña-Casanova et al., 2005). The patients with severe anxiety and depression symptoms were excluded according to the Hospital Anxiety and Depression Scale (HADS) evaluation (Zigmond and Snaith, 1983; Dunbar et al., 2000).

### Psychometric and Cognitive Assessments

A neuropsychological battery made following the recommendations of the Development of screening guidelines and criteria for predementia Alzheimer's disease study (DESCRIPA) (Visser et al., 2008) was used. Other tests were included to further investigate some particular areas of interest, such as memory and executive functions. The battery included an assessment of the following cognitive domains: memory, language, praxis, visual perception, and executive function; MMSE (Mini-Mental State Examination) was used as a global test of cognition (Folstein et al., 1975). The episodic verbal memory was assessed by means of the Free and Cued Selective Reminding Test (FCSRT) (Peña-Casanova et al., 2009a). Visual memory, constructive praxis, and visuoperceptual function were assessed through the Rey–Osterrieth Complex Figure (Peña-Casanova et al., 2009a). A short-15 items version of The Boston Naming Test (Casals-Coll et al., 2014) and Token test (De Renzi and Faglioni, 1978) were used to assess language. The executive functions were assessed using the Color and Word Stroop test (Golden and Ediciones, 2010), subtests of the WAIS battery Digit Span Forward, Digit Span Backwards and the Digit–Symbol Coding (Wechsler, 2000), and subtests of scales for outcomes in Parkinson's disease–cognition (SCOPA–cog) scale, for figure completion and squares (Martínez-Martín et al., 2009). The short-term memory was evaluated by means of the Direct Digit Span from the WAIS battery (Wechsler, 2000).

Further, all participants were evaluated with in–out test, a novel paradigm design to assess episodic memory along with a simultaneous executive task thereby interfering encoding by non-mnemonic cognitive functions (Torrealba et al., 2019). It is thought that the memory deficits underlying prodromal AD might be unveiled if simultaneous executive tasks are used to engage neuronal networks which support memory encoding in the medial temporal lobes (Tang et al., 2021).

A comprehensive overview of the cohort demographic, clinical and neuropsychometric data is summarized in Table 1.

**Table 1:** Cohort demographic, clinical and neuropsychometric data.

	**HC**	**SMC**	**MCI**
**(A) General**			
Participants (*n*)	15	9	29
Gender (M/W)	7/8	3/6	12/17
Age (range)	72.2 ± 1.8 (64–86)	69.77 ± 2.1(60–79)	74.74 ± 1.2 (65–90)
Education	7.22 ± 1.77	8.12 ± 2.03	8.04 ± 0.85
BDRS	0.20 ± 0.21	2.62 ± 1.05	4.47 ± 0.48
IADL	8.00 ± 0.00	7.62 ± 0.26	7.00 ± 0.21
HADS	6.67 ± 1.00	12.12 ± 3.30	6.90 ± 1.02
MMSE score	26.8 ± 0.8	26.4 ± 0.8	25.6 ± 0.6
βA(1–42)	710.26 ± 372.35	618.92 ± 310.78	600.66 ± 318.34
t-tau	249.57 ± 200.74a	355.39 ± 195.58ab	630.14 ± 470.41b
p181 tau	45.54 ± 31.77	57.85 ± 25.99	80.03 ± 31.17
**(B) Neuropsychological assessments**			
**Episodic verbal memory**			
*FCSRT*	13.88 ± 2.16a	12.40 ± 3.06ab	4.05 ± 3.23b
**Language**			
*BOSTON*	9.63 ± 2.55	9.50 ± 3.10	7.60 ± 1.43
*TOKEN*	32.00 ± 2.40	30.50 ± 2.89	29.36 ± 2.60
**Executive functions**			
*STROOP*	−9.38 ± 7.13	−7.57 ± 9.44	−5.26 ± 8.65
*WDSB*	4.25 ± 1.25	3.80 ± 1.62	3.60 ± 1.57
*WDSF*	5.86 ± 1.25	6.50 ± 1.90	6.25 ± 1.11
**Visuoperceptual function**			
*ROCF3*	15.66 ± 3.88a	15.20 ± 10.10ab	4.45 ± 4.45b
*ROCF30*	15.93 ± 4.30a	15.20 ± 9.27ab	2.90 ± 3.67b
**Episodic memory**			
*In-out* test (IOT)	15.34 ± 4.31a	12.16 ± 5.84ab	4.13 ± 3.13b

*Data are expressed as mean ± SD. M, men; W, women; BDRS, Blessed Dementia Rating Scale; IADL, Instrumental Activities for Daily Living; HADS, Hospital Anxiety and Depression Scale; FCSRT, Free and Cued Selective Reminding Test; BOSTON, Boston naming test; TOKEN, Token test; STROOP, Stroop test; ROCF3, Rey–Osterrieth Complex Figure 3 min; ROCF30, Rey-Osterrieth Complex Figure 30 min; WDSB, WAIS Digit Spam Backwards; WDSF, WAIS Digit Spam Forward. For each row, different letters indicate statistically significant differences with p < 0.05. βA(1–42), t-tau and phospho-tau values are expressed as pg/ml*.

**The CSF samples** the CSF samples were collected by lumbar puncture using a standard procedure to minimize the risk of biological or chemical contamination. The CSF samples were collected in sterile polypropylene tubes and centrifuged for 20 min at 2,000 *g* at room temperature. The supernatants were aliquoted into polypropylene storage tubes and frozen at −80°C until analysis.

### Determination of βA, t-Tau, and p-Tau

The concentrations of βA peptide 1–42 (Aβ), t-tau, and 181Thr-phosphorylated-tau (*p*-tau) were determined in CSF samples using appropriate Enzyme-Linked Immunosorbent Assay (ELISA)-kits (INNOTEST®, Fujirebio, Ghent, Belgium) following manufacturer's specifications.

### Determination of Specific Proteins in CSF

The CSF proteins were determined by means of indirect ELISA assays, following the protocols described in Lin (Lin, 2015), Kohl and Ascoli (Kohl and Ascoli, 2017). Briefly, a 10-μg CSF proteins were immobilized onto high protein affinity polystyrene ELISA microplates using carbonate–sodium bicarbonate buffer (0.07 M NaHCO_3_, 0.03 M Na_2_CO_3_, pH 9.6 in double-distilled water). The plates were incubated for 16–18 h under agitation (50–100 rpm) at 4°C, and then washed 3 times with 0.05% PBS–Tween20 buffer at room temperature. Subsequently, the wells were incubated with primary antibodies directed to the protein markers of the study diluted in a 5% PBS–BSA solution. The antibody incubation took place during 16–18 h at 24°C. The primary antibody was recognized by a secondary peroxidase-bound antibody (anti-rabbit or anti-mouse diluted in PBS-T). Then, secondary peroxidase-bound antibodies were oxidized using 1-Step™ Ultra TMB–ELISA (Thermo Fisher) during 30 min and the reaction was stopped by adding 1 N H_2_SO_4_. The technical information on the primary and the secondary antibodies is detailed in Supplementary Table 1. The absorbance measurements were carried out at 450 nm wavelength in a VICTORTM X5 spectrophotometer (Perkin Elmer) being the color intensity directly proportional to the amount of protein. The standard dose curve were used to establish the optimal antibody concentrations. A semiquantitative value was obtained by calculating the ratio of absorbance/total protein. As a control of specific antibody binding, other antibodies against non-specific targets of the study (i.e., amyloid precursor protein and tubulin) were used for comparison.

### Statistical Analyses

The data were initially assessed by one-way analyses of variance (ANOVA) followed by Tukey's or Games-Howell *post-hoc* tests, where appropriate. Kruskal–Wallis and Mann–Whitney *U*-tests were used in cases where normality was not achieved. The general linear models (GLM) were used to quantify main factors and to test for the presence of potential covariates. The Pearson's and partial correlation analyses as well as linear regression analyses were performed to assess the significance of bivariate relationships between different variables under study. The reliability analyses were used to test for intraclass consistencies (Cronbach's alpha) across groups. The multivariate statistics was performed using principal component analyses (PCA) and discriminant function analysis (DFA). The factor scores form PCA were compared using ANOVA-I. The predictive variables in DFA were chosen according to the number of cases in each group to fulfill the assumptions of discriminant analysis (Huberty, 1994). The multinomial logistic regression models on principal components were used as exploratory techniques to validate group differentiation, to quantify weight of classical biomarkers, and to predict subjects belonging to a particular diagnoses group.

## Results

### ER-Signalosome Proteins Are Present in the CSF of Normal Subjects and Vary Depending on the Disease Staging

**Figure 1:**
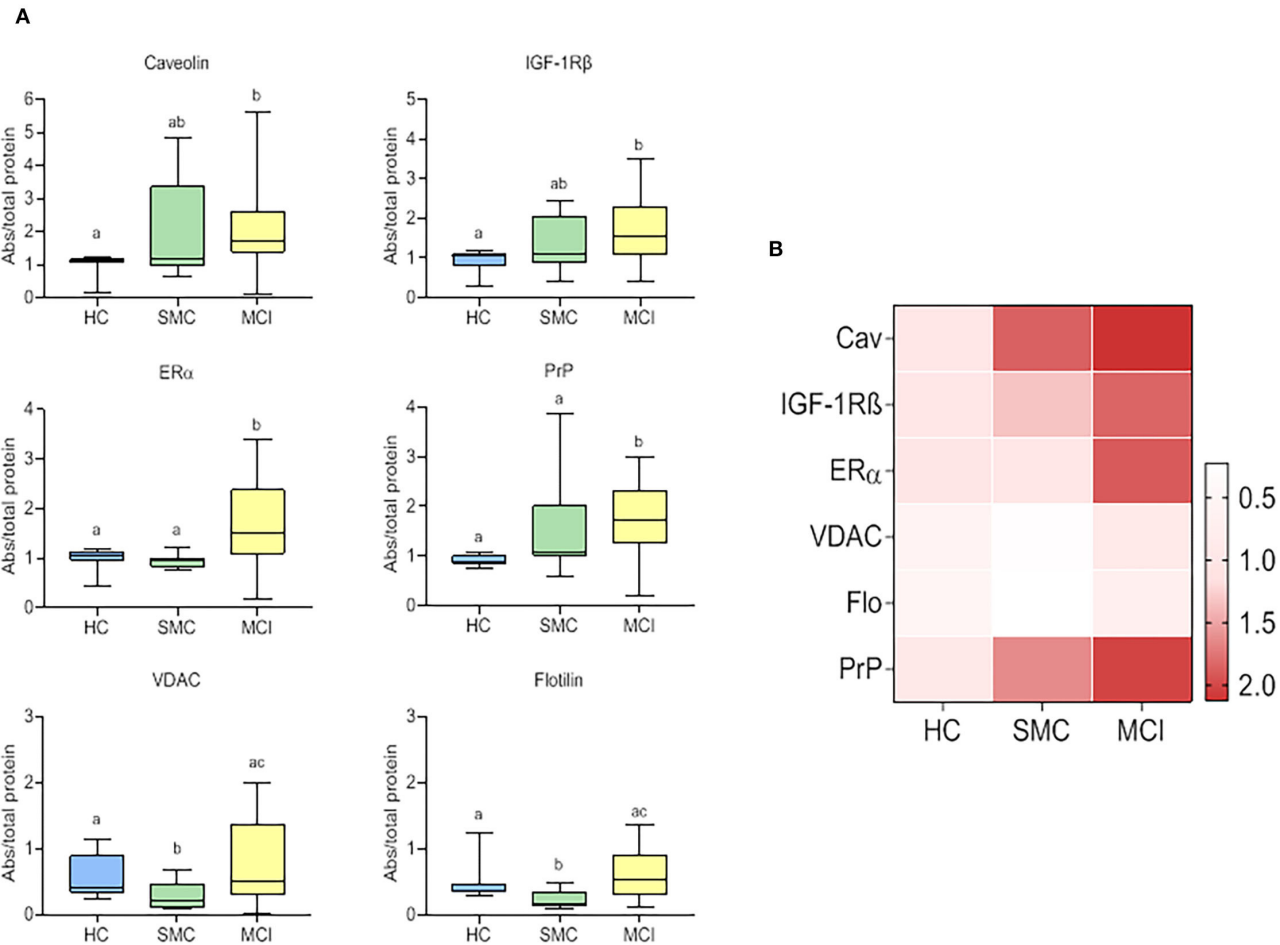
Box plots for individual signalosome protein contents in the CSF of HC, SMC, and MCI groups **(A)**. The data were submitted to one-way ANOVA or Kruskal–Wallis test followed by *post-hoc* Tukey's HSD test, Games–Howell or Mann–Withney *U*-test where appropriate. The different letters in each plot indicate statistically significant differences with *p* < 0.05. **(B)** Heatmap representation of mean protein contents in the three groups **(B)**.

All six signalosome proteins (PrPc, caveolin-1, flotillin-1, IGF1R, VDAC, and ERα), were detected in the CSF from HC. It should be emphasized that this is the first report demonstrating the presence of a set of membrane-associated signalosome proteins in the CSF of humans, and demonstrate the movement of these proteins from the brain tissue to the extracellular compartment and eventually to the CSF. The presence of such membrane components in control subjects encouraged us to seek for changes in prodromal stages of AD. The data obtained in SMC and MCI groups revealed significant differences compared to HC for most of the proteins assayed (Figure 1). In general, the protein contents were highest in MCI patients (PrPc, caveolin-1, IGF-1Rβ, VDAC, flotillin-1, and ERα) and lowest in HC subjects (PrPc, caveolin-1, IGF-1*R*β, and ERα) (Figure 1). The case of SMC was particularly different in that caveolin-1 and IGF-1Rβ levels were intermediate between HC and MCI groups, VDAC and flotillin, were significantly lower than in the other two groups, and PrPc and ERα levels were similar to HC but significantly lower than for MCI group. Taken together, these results indicate that the destabilization ER-signalosome and/or degradation of signalosome-containing membrane domains in neural cells occur early during the development of memory and cognitive decline, and that these changes might represent early molecular indicators of brain deterioration and eventually neurodegeneration.

### Bivariate Relationships Between ER-Signalosome Proteins in the CSF

**Figure 2:**
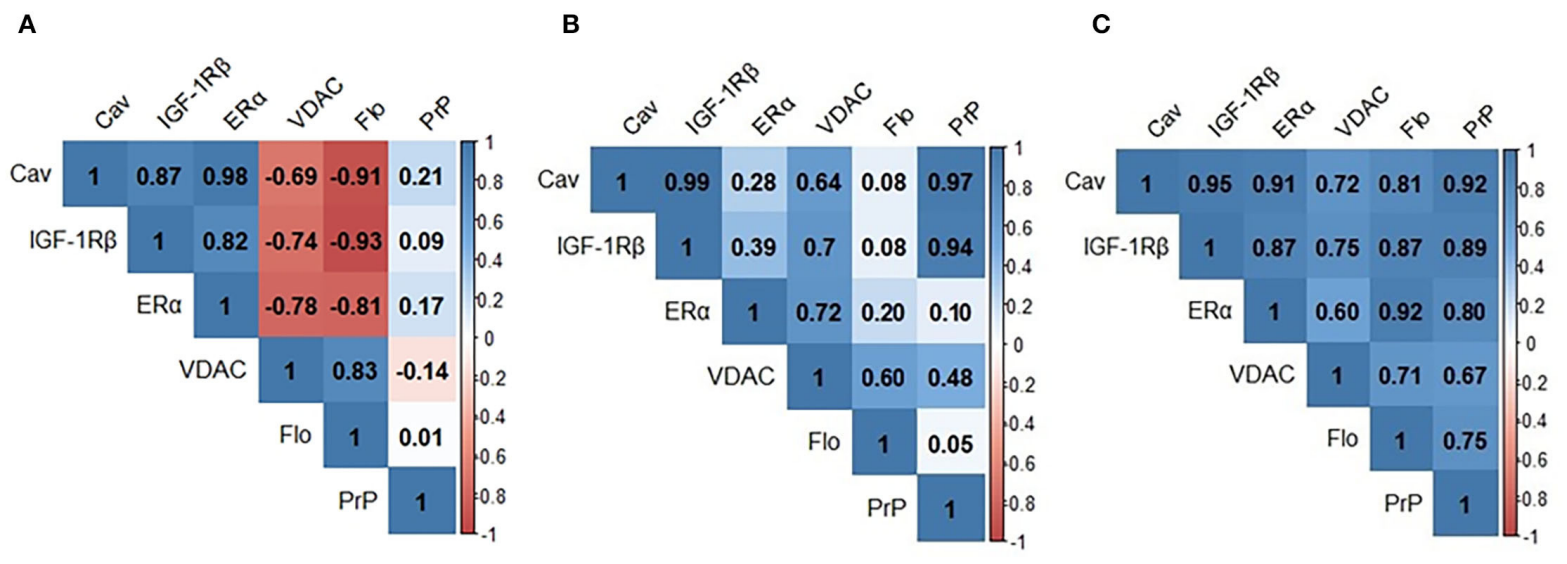
Pearson's correlation matrixes for signalosome proteins in the CSF of HC **(A)** SMC **(B)** and MCI **(C)** groups. Blue scale is used for positive correlations and red scale for negative correlations. Cav, caveolin; IGF-1Rβ, Insulin-like growth factor-1 receptor β; VDAC, voltage dependent anion channel; Flo, flotillin; PrP, Prion protein.

We next analyzed the bivariate relationships between signalosome proteins in the CSF of HC, SNC, and MCI groups. We initially performed Pearson's correlation analyses and the results are shown in Figure 2. In the HC group (Figure 2A) significant positive correlations were detected for Cav/IGF-1Rβ (*r* = 0.872; *p* < 0.001), Cav/ERα (*r* = 0.978; *p* < 0.001) and IGF-1Rβ/ERα (*r* = 0.820; *p* < 0.0010) and Flo/VDAC (*r* = 0.828; *p* < 0.001) pairs. The negative correlations were observed for VDAC/Cav (*r* = −0.695; *p* = 0.008), VDAC/IGF-1Rβ (*r* = −0.740; *p* = 0.002), VDAC/ERα (*r* = −0.781; *p* = 0.001), Flo/Cav (*r* = −0.912; *p* < 0.001), Flo/IGF-1Rβ (*r* = −0.930; *p* = 0.000), and Flo/ERα (*r* = −0.809; *p* < 0.001) pairs. In the SMC group (Figure 2B), the most existing relationships in HC group disappear and few new ones become evident: PrP/Cav (*r* = 0.951; *p* < 0.001) and PrP/IGF-1Rβ (*r* = 0.891; *p* = 0.003). Finally, for the group MCI (Figure 2C), the overall pattern indicates novel relationships affecting PrP, which were undetected in the other two groups: PrP/ERα (*r* = 0.697; *p* < 0.001), PrP/VDAC (*r* = 0.616; *p* = 0.0013) and PrP/Flo (*r* = 0.738; *p* < 0.001) and opposite correlations for some of the ratios detected in HC subjects. Thus, the ratios Flo/Cav (*r* = 0.716; *p* < 0.001), Flo/IGF-1Rβ (*r* = 0.873; *p* < 0.001) and Flo/ERα (*r* = 0,921; *p* < 0.001) which were negative in HC, but were absent in SMC and became positive in MCI subjects.

**Figure 3:**
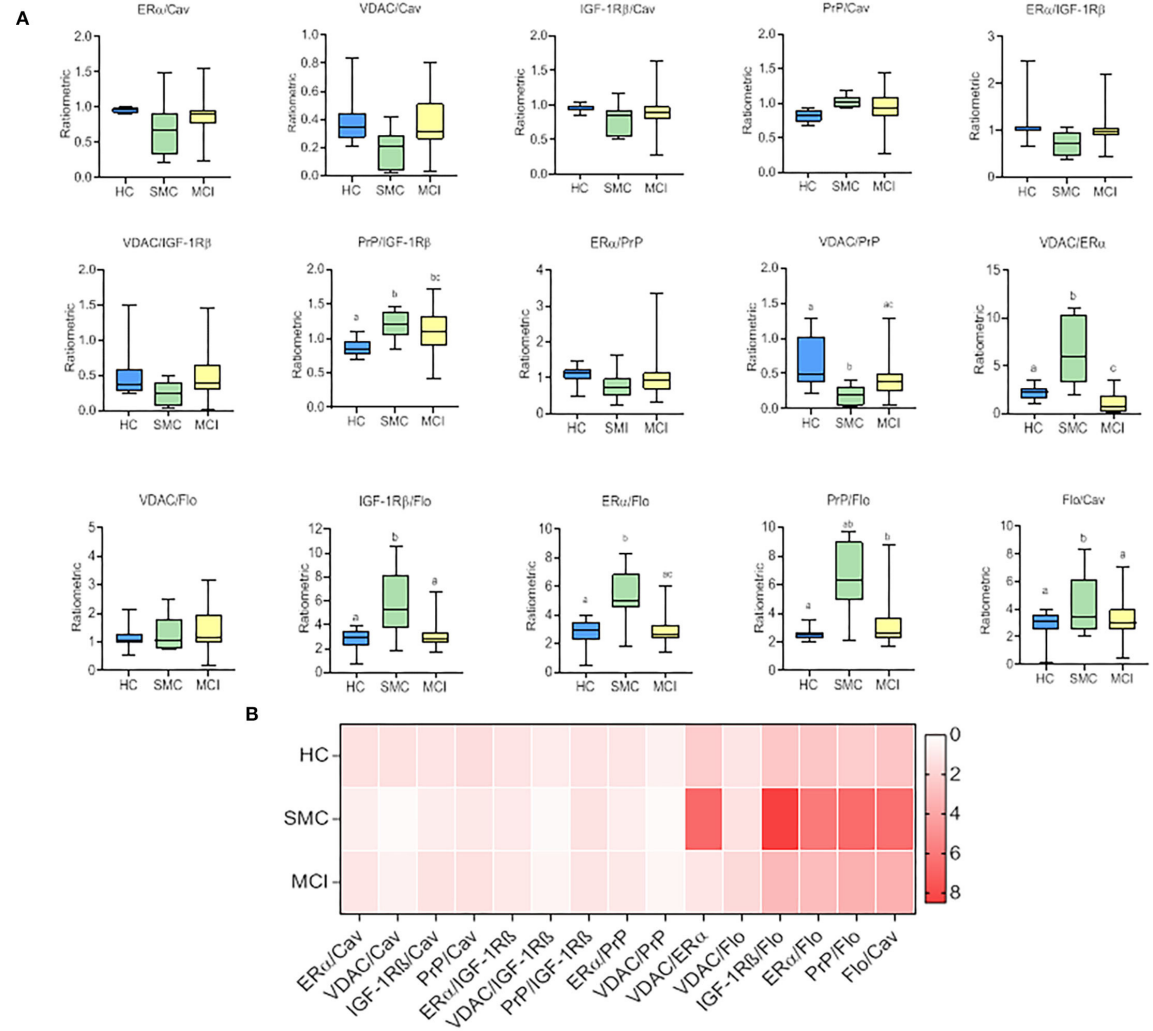
The box plots for signalosome protein ratios **(A)** in the CSF of HC, SMC and MCI groups. The data were submitted to one-way ANOVA or Kruskal–Wallis test followed by *post-hoc* Tukey's HSD test, Games–Howell or Mann–Withney *U*-test where appropriate. The different letters in each plot indicate statistically significant differences with *p* < 0.05. **(B)** Heatmap representation of mean protein ratios in the three groups **(B)**.

To evaluate whether these intragroup changes are reflected in intergroup differences, we performed one-way ANOVA on ratiometric variables. The results are shown in Figure 3 and Table 2. As it can be seen, the statistical differences between groups can be detected for PrP/IGF-1Rβ, VDAC/PrP, VDAC/ERα, IGF-1Rβ/Flo, ERα/Flo, PrP/Flo, and Flo/Cav ratios. These analyses revealed a differential ratiometric pattern for HC, SMC, and MCI groups, with the ratio VDAC/ERα ratio exhibiting total differentiation. Mean ratios and range values, along with their 95% confidence intervals for all ratios are shown in Table 2 (ratio panel). Noticeably, the ratiometric results allow a degree of definition of SMC subjects, featured by highest values of PrP/IGF-1Rβ, VDAC/ERα, IGF-1Rβ/Flo, ERα/Flo, and PrP/Flo ratios, and lowest of VDAC/PrP and Flo/Cav, probing SMC as a singular group different from HC and MCI groups.

**Table 2:** Comparative analyses of bivariate relationships between ER-signalosome proteins.

**Variable (dep/indep)**	**Parameter**	**HC**	**SMC**	**MCI**	**HC vs. SMC**	**HC vs. MCI**	**SMC vs. MCI**
ERα/Cav	Mean ± SEM	0.95 ± 0.033	0.86 ± 0.34	0.87 ± 0.22	ns	ns	ns
	Range	0.90–1.01	0.52–1.49	0.24–1.54			
	CI 95%	0.93–0.97	0.50–1.22	0.77–0.97			
	β	0.978	0.284	0.913	ns	ns	ns
VDAC/Cav	Mean ± SEM	0.39 ± 0.00	0.24 ± 0.13	0.39 ± 0.045	ns	ns	ns
	Range	0.21–0.84	0.10–0.42	0.03–0.8			
	CI 95%	0.27–0.52	0.13–0.36	0.30–0.48			
	β	−0.694	0.641	0.715	^*^	^*^	ns
IGF-1Rβ/Cav	Mean ± SEM	0.94 ± 0.05	0.88 ± 0.04	0.89 ± 0.04	ns	ns	ns
	Range	0.84–1.03	0.63–1.16	0.28–1.64			
	CI 95%	0.91–0.98	0.70–1.06	0.79–0.99			
	β	0.872	0.990	0.949	ns	ns	ns
PrP/Cav	Mean ± SEM	0.81 ± 0.03	1.02 ± 0.04	0.95 ± 0.05	^*^	ns	ns
	Range	0.67–0.93	0.92–1.18	0.28–1.6			
	CI 95%	0.75–0.87	0.92–1.11	0.84–1.07			
	β	0.211	0.971	0.923	ns	ns	ns
ERα*/*IGF-1Rβ	Mean ± SEM	1.00 ± 0.02	1.06 ± 0.27	1.01 ± 0.06	ns	ns	ns
	Range	0.89–1.2	0.45–2.35	0.43–2.18			
	CI 95%	0.95–1.05	0.37–1.76	0.88–1.14			
	β	0.820	0.390	0.872	ns	ns	ns
VDAC/IGF-1Rβ	Mean ± SEM	0.42 ± 0.05	0.29 ± 0.06	0.48 ± 0.06	ns	ns	ns
	Range	0.24–0.82	0.11–0.49	0.02–1.46			
	CI 95%	0.30–0.52	0.14–0.44	0.35–0.60			
	β	−0.740	0.699	0.752	^*^	^*^	ns
PrP/IGF-1Rβ	Mean ± SEM	0.86 ± 0.04	1.19 ± 0.09	1.11 ± 0.06	^*^	^*^	ns
	Range	0.69–1.10	0.83–1.45	0.41–1.66			
	CI 95%	0.78–0.94	0.96–1.41	0.99–1.22			
	β	0.094	0.939	0.886	ns	ns	ns
ERα/PrP	Mean ± SEM	1.18 ± 0.04	0.86 ± 0.16	0.97 ± 0.07	ns	ns	ns
	Range	0.97–1.45	0.54–1.62	0.30–2.21			
	CI 95%	1.09–1.27	0.44–1.28	0.81–1.12			
	β	0.174	0.120	0.803	ns	ns	ns
VDAC/PrP	Mean ± SEM	0.49 ± 0.07	0.25 ± 0.05	0.45 ± 0.06	^*^	ns	^*^
	Range	0.22–1.05	0.08–0.41	0.05–1.29			
	CI 95%	0.34–0.63	0.13–0.36	0.32–0.56			
	β	−0.143	0.500	0.671	^*^	^*^	ns
VDAC/ERα	Mean ± SEM	2.39 ± 0.06	4.09 ± 1.06	1.10 ± 0.21	^**^	^***^	^***^
	Range	1.13–3.97	1.94–6.18	0.1–3.58			
	CI 95%	1.89–2.88	0.71–7.45	0.66–1.56			
	β	−0.781	0.212	0.599	^*^	^*^	ns
VDAC/Flo	Mean ± SEM	1.20 ± 0.14	1.32 ± 0.30	1.36 ± 0.15	ns	ns	ns
	Range	0.52–2.15	0.80–2.48	0.09–3.16			
	CI 95%	0.90–1.50	0.47–2.16	1.06–1.66			
	β	0.828	0.602	0.602	ns	ns	ns
IGF-1Rβ/Flo	Mean ± SEM	2.97 ± 0.17	4.05 ± 1.15	2.92 ± 0.19	^***^	ns	^***^
	Range	2.13–3.86	1.76–5.24	1.73–5.42			
	CI 95%	2.60–3.34	−0.89–9.00	2.52–3.32			
	β	−0.930	0.083	0.873	ns	^*^	ns
ERα/Flo	Mean ± SEM	2.98 ± 0.16	4.02 ± 0.73	2.86 ± 0.19	^***^	ns	^**^
	Range	2.26–3.93	1.86–5.00	1.42–5.85			
	CI 95%	2.62–3.34	1.70–6.34	2.46–3.25			
	β	−0.809	0.201	0.201	ns	^*^	ns
PrP/Flo	Mean ± SEM	2.52 ± 0.11	5.74 ± 1.37	3.24 ± 0.31	^***^	^**^	ns
	Range	2.00–3.55	2.08–8.71	1.71–7.15			
	CI 95%	2.27–2.76	1.37–10.10	2.59–3.89			
	β	0.013	0.074	0.745	ns	^*^	ns
Flo/Cav	Mean ± SEM	3.13 ± 0.16	5.75 ± 1.45	3.30 ± 0.30	^***^	ns	^**^
	Range	2.36–3.97	2.02–9.05	0.66–6.55			
	CI 95%	2.77–3.46	1.15–10.35	2.67–3.93			
	β	−0.561	0.026	0.314	^*^	^*^	ns

*β, standardized regression coefficient; IC95%, 95% Confidence interval*

* *p < 0.05,*

** *p < 0.01, and*

*** *p < 0.001, respectively. ns, not significant*.

**Figure 4:**
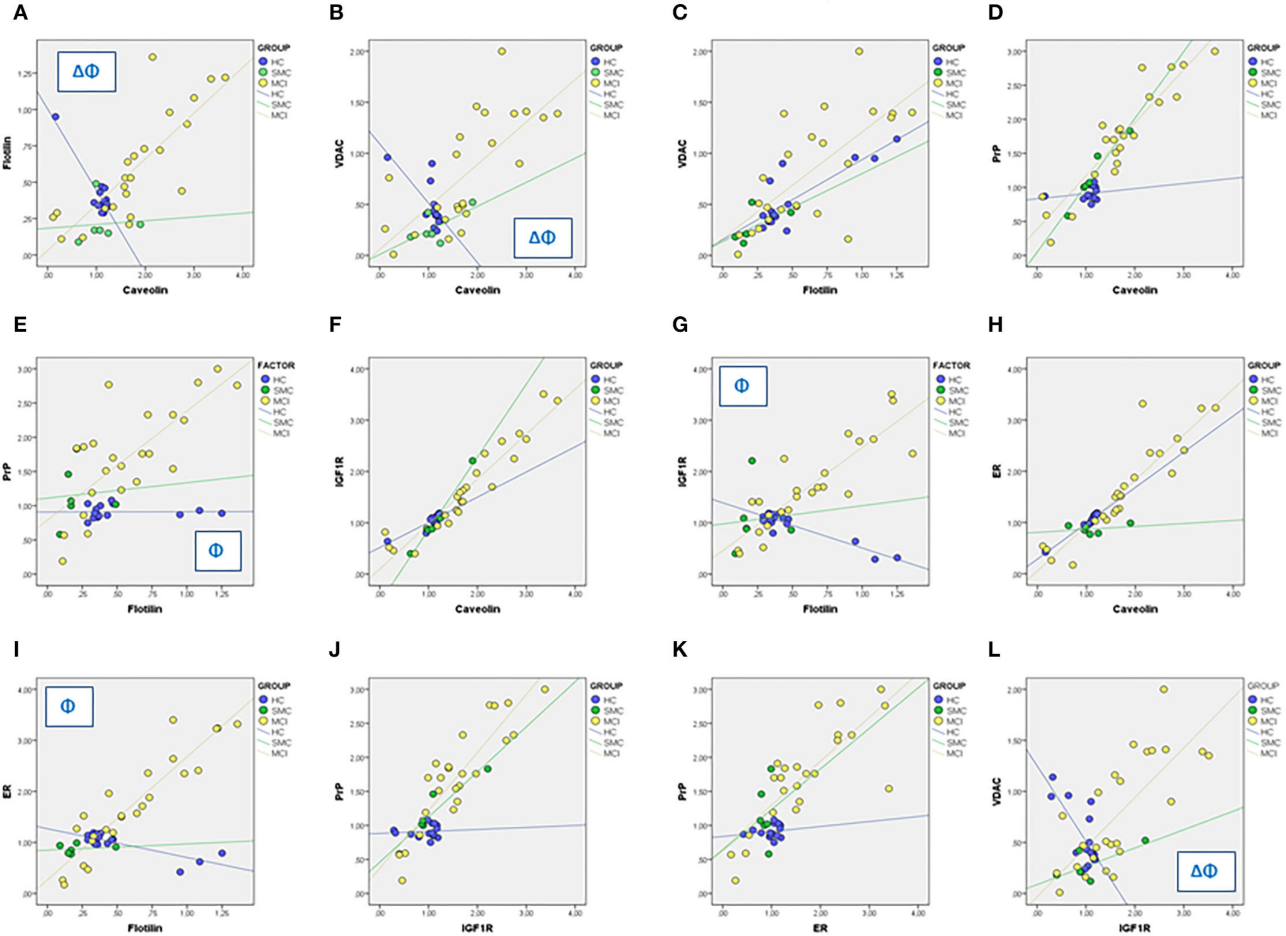
Linear regression analyses for different associations between signalosome proteins in the CSF of HC, SMC, and MCI groups. The regression coefficients and statistical significances in panels **(A–L)** are indicated in Table 2. Δ: *p* < 0.05 HC vs. SMC. Φ: *p* < 0.05 HC vs. MCI.

We next used linear regression analyses to get a deeper insight into the significance of these relationships between ER signalosome proteins. The results summarized in Table 2 (Regression coefficients, β) and Figure 4 revealed a number of bivariate relationships between signalosome proteins undergo significant changes between groups.

The first relevant observation is the opposed behavior of flotillin-caveolin along the HC→ MCI transition (Figure 4A). This observation is unprecedented and strongly suggests the remodeling of scaffold proteins in the signalosome structure. Second, the proportion of VDAC in the caveolin fraction is reduced as the amount of caveolin increases in the HC group, but augments progressively in the SMC and MCI groups (Figure 4B). The association of VDAC with flotillin is not affected in the H→ MCI transition (Figure 4C). This means that as the proportion of caveolin is reduced and the amount of flotillin increases toward MCI, the amount of VDAC associated to signalosomes increases.

Third, the amounts of PrP remain low both in caveolin and flotillin fractions in HC group, but increase significantly in both fractions in MCI subjects (Figures 4D,E). Similar results have been found for IGF-1Rβ (Figures 4F,G) and for ERα (Figures 4H,I). Of note, the associations PrP-caveolin (but not PrP-flotillin) and IGF-1Rβ-caveolin (but not IGF-1Rβ-flotillin) increase in SMC group to a similar extent (similar slope) than in MCI subjects (Figures 4D,F). The similarity of PrP, IGF-1Rβ, and ERα regression lines as function of flotillin and caveolin contents explains the collinearity of their bivariate relationships (Figures 4J,K), and suggest that these proteins are physically associated and interacting in the ER signalosomes. Noticeably, the degree of association depends on the staging, being the complexity of associations most evident in the MCI group. Finally, a particularly interesting observation is their relationship with VDAC, which is negatively related to IGF-1Rβ in HC group but become progressively more positive along the transition HC→ SMC→ MCI (Figure 4l).

The internal consistency, as determined by analyses of Cronbach's alpha coefficient, also suggests changes in the interaction between ER-signalosome proteins along the disease progression. Indeed, the results indicate that Cronbach's alpha increase along the transition HC (α = 0.057, *p* = 0.76)→ SMC (α = 0.83, *p* = 0.001)→ MCI (α = 0.96, *p* < 0.001). Notably, the internal consistency in HC group increased significantly when VDAC and flotilin were excluded from the analyses (α = 0.850, *p* < 0.001), which agrees with the hypothesis of ER-signalosome remodeling in the transition to SMC. For proteins ratios, best Cronbach's alpha is exhibited by SMC group (α = 0,832, *p* = 0.007). These novel features are interesting since they suggest very early alterations in signalosome protein associations which are reflected in the CSF, even before classical biomarkers become altered (shown in Table 1).

### Relationships Between ER-Signalosome Proteins and Classical CSF Biomarkers

The levels of classical CSF biomarkers for AD, βA (Aβ) peptide, t-tau (t-tau) and 181-Thr p-tau were determined in subjects. The results are summarized in Table 1. In general, the values are in good agreement with most studies for prodromal AD reported so far. Thus, we found particularly interesting to explore the potential association of these biomarkers with the changes observed here for ER-signalosome proteins.

First, we assessed the role of classical biomarkers as covariates for group differences in ER-signalosome protein levels using GLM. The GLMs were designed to include each ER-protein as independent variable and Aβ, t-tau, and *p*-tau as covariates (Table 3A). The classical biomarkers, included either as single variables (Aβ, *t*-tau, and *p*-tau) or combined (Aβ, t-tau, and p-tau), failed to demonstrate any significant influence as covariates nor interaction with ER proteins compared to the results shown in Figure 1 (as shown in the last column “Factor” of Table 3A), indicating that group differences in prodromal stages were independent of classical biomarkers but the disease stage. Next, we performed Pearson's correlation analyses between ER-signalosome proteins and classical biomarkers analyses. The results in Table 3B indicate poor, mostly not significant, correlation coefficients between the two set of variables in either stage groups or in whole data set. Further, we performed partial correlation analyses using βA peptide, t-tau and p-tau as independent variables controlling for changes in correlation coefficients between ER-signalosome proteins. The results shown in Table 3C revealed negligible effects of classical biomarkers (either individually or combined) on the existing correlation between any protein pair (shown in Table 3C on whole dataset for Aβ, t-tau, and p-tau). Similar results were obtained for individual groups.

Table 3Relationships between ER-signalosome proteins and classical AD biomarkers.

**Table:** 

**(A) Analyses of covariance**						
		**A** ***β***	**t-tau**	**p-tau**	**A** ***β*** **, t-tau, and p-tau**	**FACTOR (group)**
Cav	*F*_2,43_	0.560	0.002	1.362	1.150	3.872
	*p*	0.459	0.968	0.251	0.291	0.030
IGF-1R	*F*_2,43_	0.202	0.298	3.024	0.609	4.118
	*p*	0.656	0.588	0.091	0.440	0.025
ERα	*F*_2,43_	0.059	0.118	1.378	0.471	5.192
	*p*	0.809	0.733	0.248	0.497	0.010
VDAC	*F*_2,43_	1.959	0.211	1.242	0.595	5.266
	*p*	0.170	0.648	0.273	0.445	0.010
Flo	*F*_2,42_	0.237	0.233	1.711	0.295	5.223
	*p*	0.629	0.632	0.199	0.591	0.010
PrP	*F*_2,42_	0.175	0.001	2.588	1.353	6.010
	*p*	0.678	0.976	0.117	0.253	0.006

**Table:** 

**(B) Pearson's correlation analyses for HC, SMC, MCI groups, and whole dataset**													
		**HC**			**SMC**			**MCI**			**DATASET**		
		**A** ***β***	**t-tau**	**p-tau**	**A** ***β***	**t-tau**	**p-tau**	**A** ***β***	**t-tau**	**p-tau**	**A** ***β***	**t-tau**	**p-tau**
Cav	*r*	−0.107	0.024	−0.010	−0.032	−0.655	−0.788	0.056	−0.018	0.074	−0.100	0.022	0.021
	*p*	0.741	0.941	0.977	0.951	0.078	0.020	0.789	0.930	0.727	0.525	0.886	0.893
	*n*	12	12	12	6	8	8	25	25	25	43	45	45
IGF−1R	*r*	0.047	0.077	0.035	−0.090	−0.573	−0.593	0.020	−0.025	0.104	−0.109	0.066	0.123
	*p*	0.885	0.811	0.913	0.866	0.138	0.121	0.924	0.905	0.620	0.485	0.668	0.421
	*n*	12	12	12	6	8	8	25	25	25	43	45	45
ER	*r*	−0.072	−0.027	−0.049	−0.111	−0.672	−0.517	−0.013	−0.078	0.009	−0.107	0.082	0.138
	*p*	0.825	0.934	0.881	0.834	0.068	0.190	0.951	0.710	0.966	0.493	0.590	0.365
	*n*	12	12	12	6	8	8	25	25	25	43	45	45
VDAC	*r*	0.532	0.224	0.188	−0.439	0.380	0.549	0.156	−0.244	−0.184	0.108	−0.041	0.027
	*p*	0.075	0.483	0.559	0.383	0.353	0.159	0.455	0.240	0.378	0.490	0.789	0.858
	*n*	12	12	12	6	8	8	25	25	25	43	45	45
Flo	*r*	0.147	0.109	0.131	−0.290	0.568	0.514	0.040	−0.093	−0.002	0.027	0.068	0.130
	*p*	0.649	0.736	0.684	0.577	0.142	0.193	0.855	0.666	0.994	0.865	0.662	0.399
	*n*	12	12	12	6	8	8	24	24	24	42	44	44
PrP	*r*	0.014	0.241	0.291	0.129	−0.596	−0.695	−0.036	0.070	0.191	−0.216	0.128	0.148
	*p*	0.965	0.450	0.358	0.808	0.119	0.056	0.869	0.746	0.371	0.169	0.409	0.339
	*n*	12	12	12	6	8	8	24	24	24	42	44	44

**Table:** 

**(C) Pearson's and partial correlation analyses performed in whole dataset**								
			**Pearson's correlation**					
Partial correlation (Aβ, p-tau, and t-tau)			**Cav**	**IGF-1R**	**ERα**	**VDAC**	**Flo**	**PrP**
	Cav	*r*	1	0.952^**^	0.913^**^	0.682^**^	0.701^**^	0.915^**^
		*n*	43	43	43	43	42	42
	IGF-1R	*r*	0.946^**^	1	0.864^**^	0.627^**^	0.553^**^	0.883^**^
		*n*	35	46	46	46	45	45
	ERα	*r*	0.901^**^	0.903^**^	1	0.565^**^	0.684^**^	0.809^**^
		*n*	35	35	46	46	45	45
	VDAC	*r*	0.704^**^	0.759^**^	0.747^*^	1	0.755^**^	0.609^**^
		*n*	35	35	35	46	45	45
	Flo	*r*	0.666^*^	0.753^**^	0.829^**^	0.800^**^	1	0.546^*^
		*n*	35	35	35	35	45	44
	PrP	*r*	0.918^**^	0.904^**^	0.883^**^	0.762^**^	0.731^**^	1
		*n*	35	35	35	35	35	45

*Aβ, β amyloid peptide; t-tau, total-tau; p-tau, phosphorylated tau*.

* *p < 0.05,*

** *p < 0.01*.

These observations are particularly relevant to this study, since they suggest that changes in the CSF contents of signalosome proteins might occur independently of amyloid peptide generation or tau protein phosphorylation. Therefore, these results indicate that changes in ER-signalosome represent early indications of neuronal degradation along prodromal stages of the disease, well before CSF alterations of classical biomarkers allow accurate diagnosis.

### Multivariate Analyses Disclose Distinctive Fingerprints for ER-Signalosome Proteins at Preclinical Stages of AD

**Figure 5:**
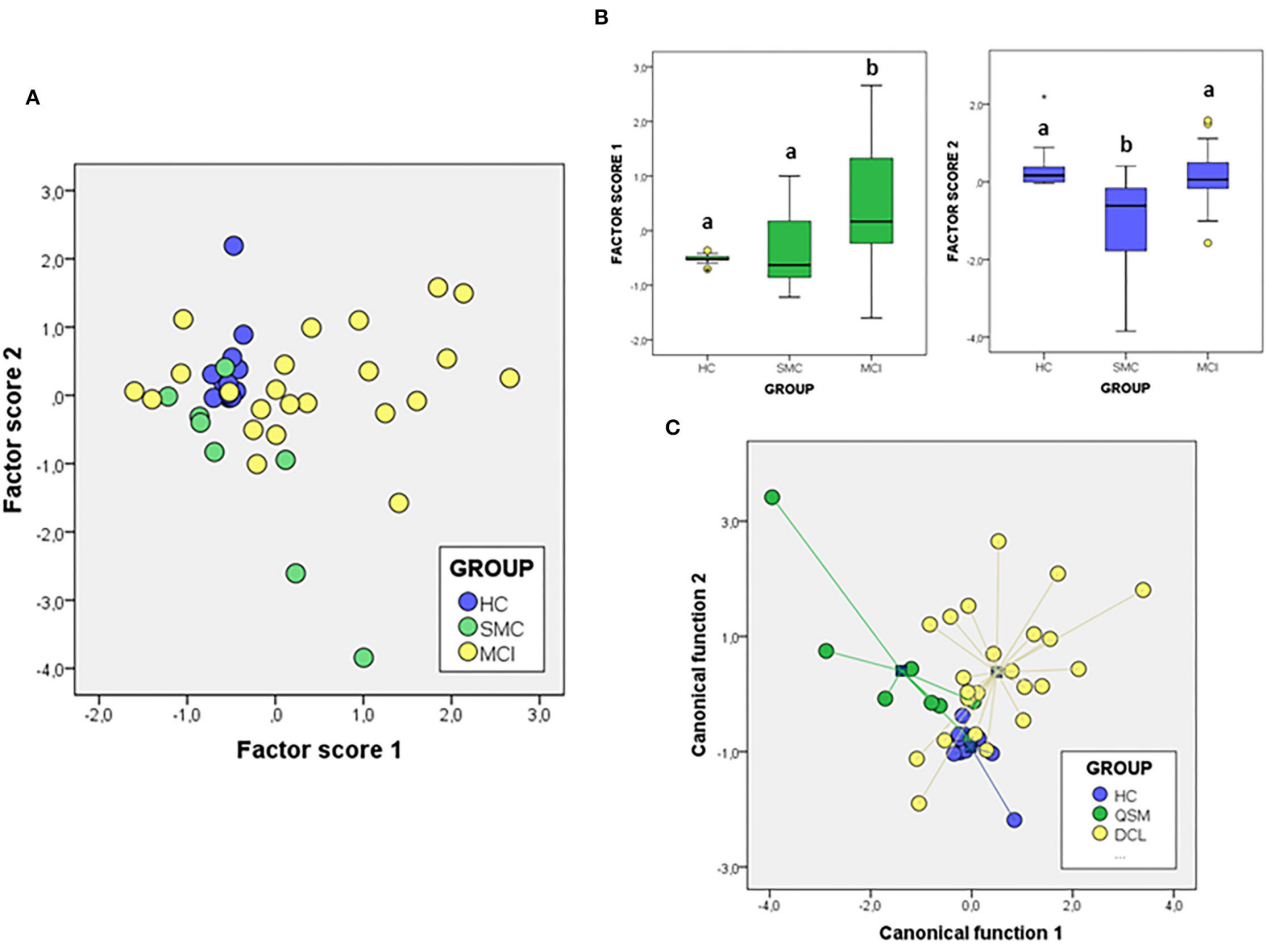
The PCA **(A,B)** and DFA **(C)** of signalosome proteins in the CSF of subjects from HC, SMC, and MCI groups. The factor scores in plot **(B)** were submitted to one-way ANOVA followed by *post-hoc* Tukey's HSD test. The different letters indicate statistically significant differences with *p* < 0.05.

We next attempted to determine the set of overall variables that might differentiate between groups. We used a variable reduction strategy based on PCA. The PCA results for signalosome proteins are illustrated in Figure 5. We found that two principal components were able to explain most overall variance (87.51%). In the component matrix, PC1 explains 70.01% of total variance while PC2 explains 17.49% of total variance. The differences between groups were mainly attributed to the variables with higher coefficients, being for PC1 ERα and insulin growth factor-1 receptor β (IGF-1Rβ) and for PC2 VDAC and caveolin. The analyses revealed that the alterations in the concentrations of signalosome-forming proteins are sufficiently different to allow a high degree of segregation between groups (Figure 5A). Further, analysis of factor scores indicates that PC1 scores differentiate HC group from MCI groups (Figure 5B). On the other hand, factor score 2 allows the statistical differentiation of SMC group from HC and MCI groups (Figure 5B).

To evaluate the probability of any patient belonging to a diagnostic group based on their CSF protein concentrations, we performed a different multivariate approach, DFA (Figure 5C). Using this analysis, it is possible to determine the quantitative influence of protein variables in the discrimination between groups, as well as the probability of each case corresponding to a particular group. The first discriminant function has a high capacity to discriminate the groups (λ_Wilks_ = 0,477, χ^2^ = 28.49, *p* < 0.005) and contains 57.6% of overall variance while the second canonical function represents the remaining 42.4% (λ_Wilk)_ = 0.724, χ^2^ = 12.40, *p* < 0.03). According to the structure coefficients, most important variables for first discriminant function are VDAC and flotillin, while for the second discriminant function, main variables are PrP and caveolin, but contributing to group discrimination to a lesser extent.

**Figure 6:**
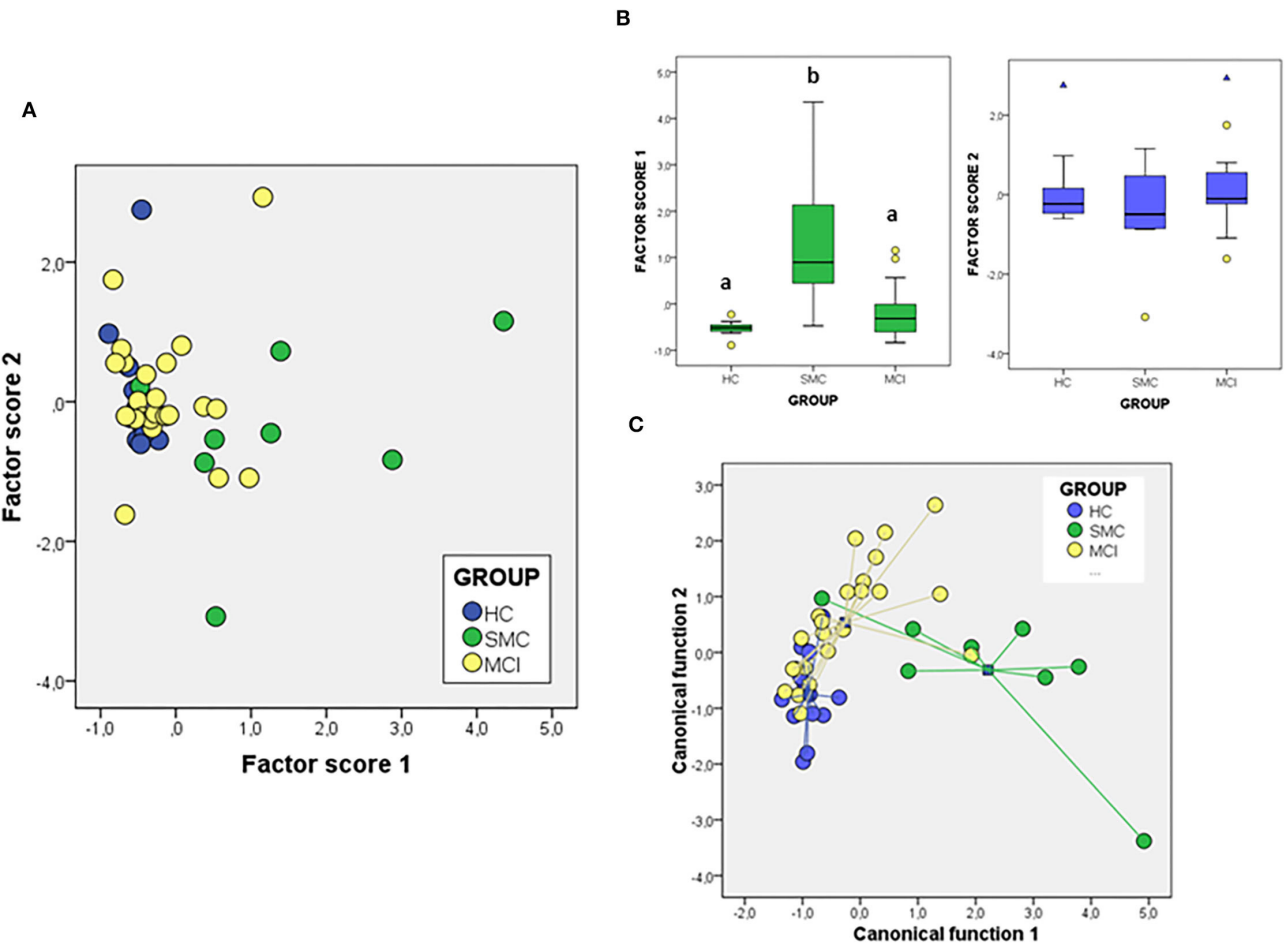
The PCA **(A,B)** and DFA **(C)** of signalosome protein ratios in the CSF of subjects from HC, SMC, and MCI groups. Factor scores in plot **(B)** were submitted to one-way ANOVA followed by *post-hoc* Tukey's HSD test. The different letters indicate statistically significant differences with *p* < 0.05.

We have also addressed whether the study of protein ratios by multivariate PCA and discriminant analysis might improve the differentiation of groups (Figure 6). In the case of the PCA analysis with the ratios obtained from the proteins, the total variance explained was 76.20%, with components 1 and 2 contributing by 56.89 and 19.13% of variance, respectively. The variables that contribute most to component 1 are the ratios IGF-1Rβ/Flo and PrP/Flo (positively) and VDAC/PrP (negatively) whiles for component 2 the ratios IGF-1Rβ/ERα (positively) and ERα/Flo (negatively) were the main variables. The analyses of factor scores obtained for each group, revealed that scores from component 1 allowed the significant differentiation of SMC group not only from HC but also from MCI (Figure 6B). No differences between groups is observed with the scores of component 2 (Figure 6B). Regarding DFA (Figure 6C), the first canonical function (λ_Wilk)_ = 0.326 χ^2^ = 41.97, *p* < 0.001) accounted for most of the overall variance (77.7%) and was mainly determined by the ratios IGF-1Rβ/Flo, PrP/Flo, and ERα/Flo, with similar potency.

To further explore the predictive value of multivariate outcomes, we performed multinomial logistic regression analysis using diagnosis (HC/SMC/MCI) as the dependent variable and principal components PC1 and PC2 from protein levels analyses describe above, as the independent variables. In this model PC1 predicted SMC and MCI diagnosis with 78.3 and 62.5% accuracy, respectively (likelihood ratio: LR χ^2^ = 28.96, *p* < 0.001). The diagnoses of MCI was heavily related to PC1 [regression coefficient: β = 1.573; Wald's χ^2^ = 5.905; *p* = 0.015; odds ratio (OR) = 4.81)], while SMC was mostly related to PC2 (β = −3.785; Wald's χ^2^ = 6.633; *p* = 0.010; OR = 0.023). Further, when the model included AD biomarkers Aβ and p-tau, and ER-signalosome parameters PC1 and PC2, predictability of MCI and SMC were 73.9 and 50.0%, respectively (LR χ^2^ = 40.66, *p* < 0.001); therefore, the slightly worse than in the absence of AD biomarkers. The model parameters confirm the weights of PC1 and PC2 for the diagnoses of SMC and MCI. However, a subtle, yet significant, contribution of Aβ could be detected for MCI group (β = −0.007; Wald's χ^2^ = 6.672; *p* = 0.010; OR = 0.993) and SMC (β = −3.785; Wald's χ^2^ = 5,879; *p* = 0.015; OR = 0.987).

### Relationships Between Neurocognitive Tests and Principal Components

**Figure 7:**
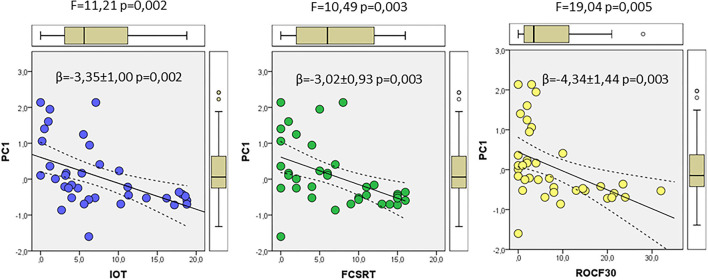
The associations of neurocognitive tests and multivariate outcomes shown in Figure 6. The linear relationships between PC1 as dependent variable and in–out (left), FCSRT (middle), and ROCF30 (right) tests as independent variables along the whole range of data are shown. The regression lines are represented along with the corresponding 95% confidence intervals and individual box-plots, in each analysis. The regression coefficients (β) and significance of regression models are indicated in each panel.

We finally used the results from PCA to evaluate potential relationships with the neurocognitive tests FCSRT, ROCF3, ROCF30, and in–out test, which showed significant differences between controls and prodromal stages, as indicated in Table 1. First, we used a correlation analyses to evaluate the association between each neurocognitive test and the factor scores from multivariate analyses of ER-signalosome described above. The results of these analyses are shown in Table 4. The four tests displayed significant negative correlations with PC1 for protein levels (PC1_P) but no with PC2 (PC2_P). Neither PC1 (PC1_R) nor PC2 (PC2_R) for protein ratios were significantly associated with neurocognitive scores (Table 4). The four cognitive tests were positively related with each other, but the best correlation with PC1_P was detected for in–out test (*r* = −0.493, *p* = 0.002). The multiple regression analyses indicate that PC1_P (β = −3.46 ± 1.11, *p* = 0.004) alone is sufficiently predictive for in–out test and that incorporation of additional components fails to improve overall fit (*F* = 3.19, *p* = 0.026). The results in Figure 7 display the regression lines for in–out test, FCSRT, and ROCF30 along with 95% confidence intervals and the box-plots for PC1_R and test punctuations. These results demonstrate an inverse relationship between neurocognitive scores and PC1_P. No further intergroup statistical analyses were performed due to the reduced number of factor scores within each GDS group.

**Table 4:** Correlation analyses for neurocognitive tests and principal components from Figures 6, 7.

		**FCSRT**	**IOT**	**ROCF3**	**ROCF30**	**PC1_P**	**PC2_P**	**PC1_R**	**PC2_R**
FCSRT	*r*	1	1.886^**^	1.784^**^	1.812^**^	−0.48^**^	0.095	−0.086	0.063
	*p*		0.000	0.000	0.000	0.003	0.575	0.595	0.695
	*n*	45	45	45	45	37	37	41	41
IOT	*r*	0.886^**^	1	0.792^**^	0.822^**^	−0.49^**^	0.030	−0.010	0.199
	*p*	0.000		0.000	0.000	0.002	0.858	0.949	0.213
	*n*	45	45	45	45	37	37	41	41
ROCF3	*r*	0.784^**^	0.792^**^	1	0.956^**^	−0.44^**^	0.076	0.001	0.023
	*p*	0.000	0.000		0.000	0.006	0.657	0.996	0.886
	*n*	45	45	45	45	37	37	41	41
ROCF30	*r*	0.812^**^	0.822^**^	0.956^**^	1	−0.45^**^	0.106	−0.042	0.082
	*p*	0.000	0.000	0.000		0.005	0.531	0.794	0.612
	*n*	45	45	45	45	37	37	41	41
PC1_P	*r*	−0.480^**^	−0.49^**^	−0.440^**^	−0.453^**^	1	0.271	−0.039	0.154
	*p*	0.003	0.002	0.006	0.005		0.109	0.818	0.364
	*n*	37	37	37	37	37	36	37	37
PC2_P	*r*	0.095	0.030	0.076	0.106	0.271	1	−0.82^**^	0.119
	*p*	0.575	0.858	0.657	0.531	0.109		0.000	0.483
	*n*	37	37	37	37	36	37	37	37
PC1_R	*r*	−0.086	−0.010	0.001	−0.042	−0.039	−0.82^**^	1	0.018
	*p*	0.595	0.949	0.996	0.794	0.818	0.000		0.910
	*n*	41	41	41	41	37	37	41	41
PC2_R	*r*	0.063	0.199	0.023	0.082	0.154	0.119	0.018	1
	*p*	0.695	0.213	0.886	0.612	0.364	0.483	0.910	
	*n*	41	41	41	41	37	37	41	41

*PC1_P, Factor scores from principal component 1 for protein levels in Figure 6; PC2_P: Factor scores from principal 2 for protein levels in Figure 7; PC1_R, Factor scores from principal component 1 for protein ratios in Figure 7; PC2_R, Factor scores from principal 2 for protein ratios in Figure 6*.

** *p < 0.01. r: Pearson's correlation coefficient*.

## Discussion

The most recently identified multimolecular signalosome in nerve cells is the ER-signalosome, a complex multimolecular cluster of proteins integrated in lipid rafts and involved in neuronal preservation (Ramírez et al., 2009; Marin, 2011; Marin et al., 2012; Marin and Diaz, 2018). Furthermore, current evidence indicates that impairment of this particular signalosome is linked to the development of neurodegenerative diseases, in particular to AD (Marin et al., 2009, 2012; Marin, 2011; Canerina-Amaro et al., 2017; Marin and Diaz, 2018). The present study provides the first evidence for a signature of ER-signalosome alterations in the CSF at early stages of AD. Indeed, we demonstrate here that main representative components of ER-signalosome, namely ERα, IGF-1Rβ, PrP, Flot, and Cav are present in the CSF of all subjects in the analyzed cohort. Assuming that ER-signalosome proteins in the CSF reflect changes in the cortical tissue, it may be envisaged that quantitative associations between these proteins are progressively modified as the cognitive decline occurs. First, signalosome proteins (ERα, IGF-1Rβ, PrP, and Cav) exhibit display a linear trend to increase along the HC→ MCI transition (Figure 1). These observations indicate that a progressive degradation of ER-signalosome occurs in nerve cells in parallel to development of cognitive complaints.

An essential component in the ER-signalosome is caveolin-1, which functions as scaffolding protein. Some recent reports have already related caveolin-1 to cognitive impairment (Tang et al., 2021), and more interestingly, that caveoin-1 might provide the potential link for type 2 diabetes and AD co-occurrence in pathological aging (Surguchov, 2020). The caveolin-1 plays a pivotal anchoring role for the interaction of ERα/IGF-1Rβ/VDAC complex in the ER-signalosome (Marin et al., 2008; Ramírez et al., 2009). Further, it has been observed that ER-signalosome proteins associations are altered in AD frontal cortex, with a displacement of both ERα and IGF-1Rβ outside the lipid rafts as a consequence of their caveolin-1 dissociation (Canerina-Amaro et al., 2017). The significantly increased level of Cav in the CSF of MCI group is indicative that this protein is being excluded from ER-signalosome. Moreover, a clear trend exists to increase CSF Cav levels in the SMC group, which suggest that the destabilization process is progressive during these early stages of AD. This hypothesis is reinforced by the observation that caveolin-associated proteins ERα, PrP, and IGF-1Rβ are also increased in MCI patients. In addition, mismatched ERα/IGF-1Rβ/caveolin-1 association causes the redistribution of VDAC outside lipid rafts (Canerina-Amaro et al., 2017; Marin and Diaz, 2018). Indeed, it has been suggested that dissociation from ERα favors the progressive dephosphorylation of pl-VDAC and its proapoptotic activation. This promotes its physical association with βA, which results in increased neuronal vulnerability (Herrera et al., 2011a; Fernandez-Echevarria et al., 2014; Smilansky et al., 2015; Thinnes, 2015a,b; Canerina-Amaro et al., 2017). This suggests that estrogen binding to ERα signalosome may be part of the mechanisms of neuroprotection against Aβ production, and that progressive VDAC dephosphorylation and trafficking out of raft microdomains may be part of the neurotoxic mechanism that exacerbates AD progression. However, it still remains to be established whether these alterations in the ER-signalosome may also occur, and to what extent, in the brain cortex in prodromal stages of AD. Noticeably, ER-signalosome disarrangements similar to those observed in AD brains have been observed in postmortem postmenopausal women brain cortex, a finding that has been associated to the decline of estrogen production (Marin et al., 2005, 2008; Lan et al., 2015; Canerina-Amaro et al., 2017; Marin and Diaz, 2018). In this sense, it is suggestive the scenario that ER-signalosome might represent a decision step in nerve cell fate, as hypothesized by George and Wu (2012) for lipid rafts as floating islands of death and survival.

Based on the bivariate analyses performed here, it becomes clear that protein associations vary depending on the stage. Indeed, the relationship between Cav and Flo is negative in HC, inexistent in SMC and positive in MCI, which reflect a profound alteration in the scaffold structure of the ER-signalosome along HC→ MCI transition. Also, ERα, IGF-1Rβ and PrP are positively associated with Cav in all groups, but their association with Flo depends on the stage, being always negative in HC and progressively more positive in the SMC to MCI groups. This suggests a remodeling of interactions with scaffold proteins as Flo becomes more abundant. In agreement, a recent report by Abdullah and coworkers (2019) has suggested that flotillin might be a novel diagnostic marker of AD.

The highest degree of interaction of ER-signalosome proteins occurs in the MCI stage, as suggested by the very significant positive collinearity between ERα/IGF-1Rβ/PrP/Flo/Cav and the high internal consistency. In this stage, pl-VDAC appears as associated with Cav, which differs from HC subjects, where the channel is associated to Flo but excluded for its association with Cav. Thus, according to our present results, pl-VDAC activation would involve dissociation from ERα and Cav, therefore escaping from the control by ERα, at the time that its association with Flo increases. Additional studies in postmortem MCI brains would allow verifying the consistency of this hypothesis. Further, it would be particularly interesting determining the phosphorylation status of pl-VDAC in the CSF along the HC→ MCI transition, since it is known that in human hippocampus and frontal and entorhinal cortices, the porin is phosphorylated in three tyrosine residues in control brains but undergo progressive dephosphorylation correlated with the severity of the disease (Herrera et al., 2011a; Fernandez-Echevarria et al., 2014).

A relevant outcome from our bivariate and multivariate analyses is the systematic intergroup variations exhibited by different sets of proteins as well as their ratios, which allow the segregation of HC, SMC, and MCI groups. From the point of view of diagnosis this feature is very promising, since the procedure allows identification of MCI and SMC groups as singular and differentiated from previous stages (SMC and HC, respectively) and subsequent stages (MCI and AD, respectively) in the spectrum of AD. Noteworthy, the multivariate approach used here allow a separation of prodromal groups which cannot be reached by standard neurocognitive assessments. Indeed, as summarized in Table 1B, HC and SMC groups are practically undistinguishable between each other, yet they both differ from MCI stage.

Noticeably, an important conclusion from these findings is that the dynamics of ER-signalosome protein rearrangement differ, and is independent, from that of the formation of the classical histopathological markers of the disease, that is, senile plaques and neurofibrillary tangles. In fact, we demonstrate no significant influences of amyloid peptide, tau protein or p-tau, on the dynamic of ER-signalosome proteins along stages, which strongly suggest that the changes in the levels of these proteins in the CSF during prodromal stages represent another perspective of AD pathogenesis.

Although the underlying mechanisms to the ER-signalosome destabilization remain unknown, these changes apparently correlate with alterations in cell membrane microdomains such lipid rafts and perhaps other non-raft domains. Indeed, compelling evidence indicate that anomalies in lipid rafts occur in neurodegenerative diseases, including AD and Parkinson's diseases (Rushworth and Hooper, 2010; Vetrivel and Thinakaran, 2010; Fabelo et al., 2011, 2014; Hicks et al., 2012; Díaz et al., 2015; Egawa et al., 2016; Canerina-Amaro et al., 2017; Díaz and Marin, 2021). Previous studies have demonstrated changes in the lipid matrix of lipid raft and this appears to be a crucial early event in the development of AD (Fabelo et al., 2012, 2014; Díaz et al., 2015, 2018). In addition, these changes affect the physicochemical properties of the lipid rafts that can subsequently alter the protein clusters integrated in these domains (Levental et al., 2010; Diaz et al., 2012; Díaz et al., 2018; Egawa et al., 2016; Santos et al., 2016).

Indeed, even minor alterations in lipid rafts can trigger pathological effects by modifying the way lipids and proteins interact, thereby affecting signal processing and consequently nerve cell responses (Michel and Bakovic, 2007; Fabelo et al., 2014; Díaz et al., 2018; Díaz and Marin, 2021). In this sense, a plausible hypothesis is that the altered membrane lipid microenvironment may determine the trafficking of signalosome proteins from the cerebral matrix to CSF being its composition a fingerprint of the degenerative process that takes place in the brain matrix.

Finally, we have assessed the potential relationship of CSF multivariate outcomes with the neuropsychological evaluation of this cohort. Statistically significant results were obtained for in–out test, which assesses episodic memory (Torrealba et al., 2019), FCSRT which assess episodic verbal memory (Buschke, 1984; Peña-Casanova et al., 2009a), ROCF3 and ROCF30 (Rey-Osterrieth Complex Figure 3 and 30 min), which evaluates visual memory and visuoperceptual function (Peña-Casanova et al., 2009a). The in–out test is a recent neurocognitive test particularly interesting since it may detect prodromal AD with a higher degree of accuracy than conventional hippocampal-based memory tests, though avoiding reliance on executive function, which may compensate for damaged memory networks. Further, this new paradigm has a high predicting capacity in which patients with MCI will go on to develop AD (Torrealba et al., 2019).

We found that in–out test, FCSRT and ROCF30 punctuations were all negatively related to principal component 1 for CSF proteins. These results are outstanding as they indicate that CSF variables included in PC1 associate with cognitive alterations, specifically in episodic memory, and more interestingly, in predicting conversion to dementia (Torrealba et al., 2019).

A clear limitation of this study is the small cohort size used in our analyses. Unfortunately, age at onset, gender and APOE genotype could not be used as covariates in the analyses, because such information was either lacking or the access to CSF samples was limited, especially for control subjects. Nevertheless, we reckon this initial study is relevant as it highlights the involvement of alternative mechanisms underlying AD which are altered during the early development of the disease. Further longitudinal studies with larger sample sizes will be necessary to confirm the diagnostic usefulness and specificity of these novel biomarkers.

## Conclusions

This study provides the first evidence of the ER-signalosome protein alterations detectable in the CSF of prodromal stages of AD. These modifications in protein contents and their ratios indicate the progressive rearrangement of lipid raft resident ER-signalosome during the preclinical phases of AD, which may ultimately contribute to cognitive impairments. Furthermore, the changes in these CSF proteins may pave the way for the development of early biomarkers for the diagnosis of initial stages of AD. Moreover, this work can prove valuable for better understanding the early pathogenicity of AD, which might be useful in the patient stratification for clinical trials and therapy development. Additional studies on the dynamics of protein/protein interactions in lipid raft resident signalosomes during early stages of AD would be worthy of pursuing, as a method to search for novel accurate biomarkers but also for identification of molecular targets for alternative therapeutic approaches.

## Data Availability Statement

The original contributions presented in the study are included in the article/Supplementary Material, further inquiries can be directed to the corresponding author.

## Ethics Statement

The studies involving human participants were reviewed and approved by Ethical Committee of Hospital Universitario de Gran Canaria Dr. Negrin. The patients/participants provided their written informed consent to participate in this study.

## Author Contributions

MD and RM: design and conceptualized study. ET: sample collection. FM-H and RM: data analysis. FM-H, MD, and RM: interpretation of the data. FM-H, GS, and MD: statistical analyses. FM-H and MD: drafting of the manuscript. FM-H, GS, RM, and ET: revision of the manuscript. All authors read and approved the final manuscript, contributed to the article and approved the submitted version.

## Funding

This study was supported by Grants ProID2020010075 from Agencia Canaria de Investigación, Innovación y Sociedad de la Información (ACIISI, Gobierno de Canarias, Spain) and SAF2017-84454-R (MINECO, Spain). FM-H wishes to thank Fundación La Caixa–Caja Canarias (Spain) for financing her predoctoral fellowship.

## Conflict of Interest

The authors declare that the research was conducted in the absence of any commercial or financial relationships that could be construed as a potential conflict of interest.

## Publisher's Note

All claims expressed in this article are solely those of the authors and do not necessarily represent those of their affiliated organizations, or those of the publisher, the editors and the reviewers. Any product that may be evaluated in this article, or claim that may be made by its manufacturer, is not guaranteed or endorsed by the publisher.

## Supplementary Material

Supplementary Table 1 Primary and secondary antibodies used in this study.
