# Supplementary material for: Neuronal ER-Signalosome Proteins as Early Biomarkers in Prodromal Alzheimer's Disease Independent of Amyloid-β Production and Tau Phosphorylation
Source: Front Mol Neurosci. 2022 May 5;15:879146. doi: 10.3389/fnmol.2022.879146 (PMC9119323; doi:10.3389/fnmol.2022.879146)
Supplement: Supplementary Table 1 — Primary and secondary antibodies used in this study. [file Data_Sheet_1.PDF]

**Primary antibody**	**Dilution**	**Host**	**Reference**	**Supplier**
Anti-Estrogen Receptor alpha	1:30	mouse	sc-8002	Santa Cruz Biotechnology (California, USA)
Anti-IGF-1R Antibody C20	1:30	Rabbit	sc-713	Santa Cruz Biotechnology (California, USA)
Anti-Caveolin-1 Antibody	1:30	mouse	sc-53564	Santa Cruz Biotechnology (California, USA)
Anti-Flotillin-1 Antibody	1:30	mouse	sc-74566	Santa Cruz Biotechnology (California, USA)
Anti-VDAC1/Porin Antibody	1:30	Mouse	sc-390996	Santa Cruz Biotechnology (California, USA)
Anti-PrP Antibody	1:30	Mouse	sc-47730	Santa Cruz Biotechnology (California, USA)
**Secondary antibody**	**Dilution**	**Host**	**Reference**	**Supplier**
Goat Anti-Mouse IgG H&L (HRP)	1:100000	mouse	ab6789	Abcam (Cambridge UK)
Goat Anti-Rabbit IgG H&L (HRP)	1:120000	rabbit	ab6721	Abcam (Cambridge UK)
